# Polyamine Metabolism and the DHPS/eIF5A Hypusination Axis: From Metabolic Reprogramming to a Therapeutic Achilles’ Heel in Melanoma

**DOI:** 10.3390/biom16040574

**Published:** 2026-04-13

**Authors:** Kai-Li Liu, Shuo Zhang, Feng-Shuo Li, Min-Jin Chen, Yuan-Yuan Chen, Ning Zhang, Kai Wang

**Affiliations:** College of Medical Engineering, Jining Medical University, Jining 272067, China

**Keywords:** polyamine metabolism, hypusination, deoxyhypusine synthase (DHPS), eukaryotic initiation factor 5A (eIF5A), melanoma metastasis

## Abstract

The polyamine metabolic pathway, an evolutionarily conserved nexus integrating nutrient sensing, translation control, and cellular proliferation, is fundamentally rewired in cancer. Melanoma, a malignancy of melanocytes notorious for its metastatic propensity and therapy resistance, exhibits a profound dependency on this pathway, extending beyond mere polyamine abundance to the specialized function of their derivative, hypusine. This review synthesizes cutting-edge insights into the deoxyhypusine synthase (DHPS)/eukaryotic initiation factor 5A (eIF5A) hypusination circuit as a critical amplifier of oncogenic signaling in melanoma. We dissect its role as a translational rheostat for pro-tumorigenic proteomes, a driver of phenotypic plasticity underpinning invasion and vasculogenic mimicry, and a modulator of the immunosuppressive tumor microenvironment. Moving beyond the classical inhibitor GC7, we explore the emergence of novel allosteric DHPS inhibitors with compelling preclinical efficacy. Finally, we propose a paradigm shift: targeting the DHPS/eIF5A axis represents a strategy to disrupt the “non-oncogene addiction” of melanoma—its reliance on hyperactive translation and adaptive survival mechanisms—offering a promising avenue alongside targeted therapies and immunotherapies.

## 1. Introduction: Polyamines—From Housekeeping Cations to Oncogenic Effectors

Polyamines (putrescine, spermidine, spermine) are ubiquitous organic polycations [1,2,3], long recognized for their pleiotropic roles in nucleic acid stabilization [4,5], membrane integrity [6,7], and ion channel regulation [8,9]. In oncology, their status has evolved from generic proliferation markers to central players in metabolic transformation [10,11]. The frequent amplification of the MYC oncogene, a master transcriptional regulator of polyamine biosynthetic enzymes like ornithine decarboxylase (ODC) [12,13,14], creates a permissive metabolic landscape across cancers [15,16]. However, the mere accumulation of polyamines is only part of the story. A pivotal and more selective output of polyamine flux, particularly spermidine, is the post-translational hypusination of eIF5A-a modification as specific as it is essential [17,18,19,20]. This review argues that in melanoma, a cancer characterized by high mutational burden [21,22], metabolic adaptability [23,24], and robust stress response pathways [25,26], the DHPS/eIF5A axis is co-opted to meet the extraordinary translational demands of tumorigenesis, metastasis, and immune evasion, making it a target of exceptional vulnerability [27,28,29,30].

## 2. The Hypusine Circuit: A Unique Translational Control Node

### 2.1. The Biochemistry of a Two-Step “Tag”

**Figure 1:**
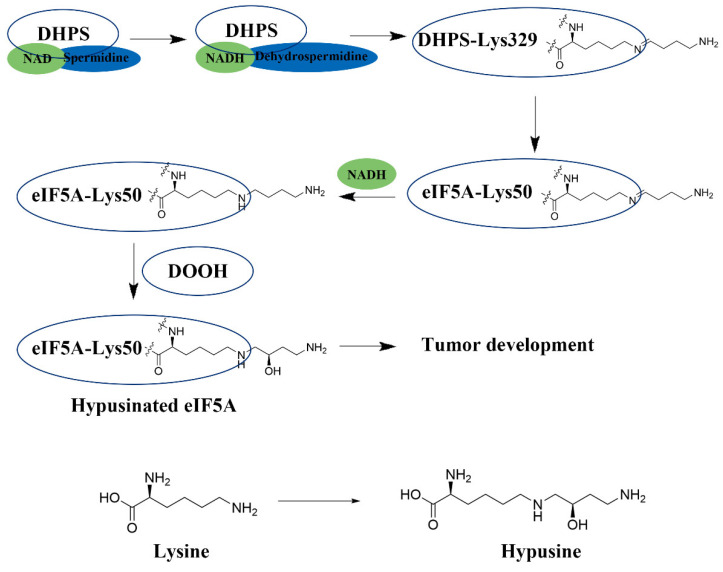
Schematic representation of the eIF5A maturation process catalyzed by DHPS and DOOH.

Hypusination is arguably biology’s most specific protein modification [31,32]. DHPS catalyzes the NAD-dependent transfer of the 4-aminobutyl moiety from spermidine to Lys50 of the eIF5A precursor, forming deoxyhypusine [33,34]. Deoxyhypusine hydroxylase (DOHH), a non-heme di-iron enzyme, then completes the synthesis of hypusine [35,36]. This irreversible modification is exclusive to two paralogs, eIF5A1 and eIF5A2, creating a direct molecular link between polyamine pools and the functional status of this translation factor [37,38] (Figure 1).

### 2.2. Beyond Initiation: eIF5A as the Elongation Specialist

Early studies mischaracterized eIF5A as an initiation factor [39,40,41]. It is now established as a crucial elongation factor that alleviates ribosome stalling at problematic sequences, notably consecutive proline residues, which pose a kinetic challenge to the peptidyl transferase center [42,43,44,45]. Thus, hypusinated eIF5A acts as a sequence-specific translator, disproportionately affecting the synthesis of proteomes enriched in polyproline motifs [46,47].

Mechanistically, hypusinated eIF5A promotes translation elongation by facilitating peptide bond formation at ribosome-stalling sequences, particularly those containing consecutive proline residues. Proline, due to its rigid cyclic structure and lack of a free amide hydrogen, acts as a poor substrate for the peptidyl transferase center, causing ribosome pausing during elongation [47]. The hypusine residue—a modified lysine with an extended, flexible 4-aminobutyl-hydroxybutyl side chain—is critical for this function. Structural studies indicate that the hypusine side chain protrudes from eIF5A into the ribosome’s E (exit) site and peptidyl transferase center, where it stabilizes the positioning of the peptidyl-tRNA and facilitates the nucleophilic attack necessary for peptide bond formation [48]. This “ribosome rescue” function is particularly essential for the translation of proteins with polyproline stretches, but emerging evidence suggests eIF5A also alleviates stalling at other problematic sequences, exerting a global effect on translation elongation efficiency [43,45].

Bioinformatic analyses reveal that such proteins are overrepresented in pathways governing cytoskeleton dynamics (e.g., ACTN4, VASP) [49], extracellular matrix remodeling (e.g., MMPs) [50], and cell cycle control [27,51] all processes central to malignancy.

### 2.3. A Nexus for Oncogenic Signaling Integration

The DHPS/eIF5A hypusination axis does not function in isolation, it is intricately embedded within and regulated by core oncogenic signaling networks, acting as both a recipient of upstream signals and a critical effector that reprograms the translational output to support malignant phenotypes [52,53]. Its position at this crossroads allows it to integrate metabolic status and growth factor cues to prioritize the synthesis of a malignancy-adaptive proteome.

#### 2.3.1. The MYC-ODC-eIF5A Positive Feedback Loop

The transcription factor MYC is a master regulator that drives the expression of ODC, the rate-limiting enzyme in polyamine biosynthesis. This leads to elevated spermidine pools, which fuel the hypusination of eIF5A [12]. Crucially, hypusinated eIF5A is not a passive beneficiary but an active facilitator of MYC-driven oncogenesis [18]. It directly regulates MYC biosynthesis at the translational level by alleviating ribosome stalling at specific, problematic motifs within the MYC mRNA coding sequence, thereby ensuring efficient MYC protein synthesis. This establishes a powerful feed-forward loop: MYC drives the transcriptional upregulation of ODC, leading to increased spermidine production. This elevated spermidine pool fuels the DHPS-mediated hypusination of eIF5A. Hypusinated eIF5A, in turn, directly facilitates the translational elongation of MYC mRNA, thereby completing a positive feed-forward loop that robustly sustains MYC protein synthesis and oncogenic activity. This circuit is a key vulnerability, as combined inhibition of ODC [54,55] (e.g., with DFMO) and eIF5A hypusination [56,57] (e.g., with GC7) synergistically disrupts this loop, leading to profound suppression of MYC protein levels, inhibition of cell proliferation, and induction of apoptosis, as demonstrated in colorectal cancer models [20].

#### 2.3.2. Regulation by the MAPK Pathway: Kinase-Dependent and Independent Mechanisms

The BRAF/MEK/ERK pathway, frequently hyperactivated in melanoma, exerts a dual-layer control over the hypusination axis [58,59]. Firstly, ERK1/2 kinase activity can transcriptionally upregulate the expression of components like DHPS and eIF5A [60]. Secondly, and more dynamically, ERK1/2 proteins themselves directly interact with DHPS in a kinase-activity-independent manner [60]. Structural studies reveal that ERK2 binding to DHPS can physically hinder the access of the eIF5A substrate to the enzyme’s active site, thereby inhibiting hypusination. Activation of the Raf/MEK/ERK cascade leads to decreased ERK-DHPS interaction and a concomitant increase in DHPS-eIF5A association, promoting hypusination. This non-canonical, scaffolding function of ERK adds a rapid, post-translational layer of regulation, allowing the cell to fine-tune hypusination rates directly in response to mitogenic signals [60].

From a melanoma genetics perspective, the functional implications of this scaffolding interaction may vary depending on the specific driver mutation. In BRAF-mutant melanomas (particularly BRAF V600E), the MAPK pathway is constitutively activated, leading to sustained ERK phosphorylation and a predicted decrease in ERK-DHPS binding, thereby relieving the inhibitory constraint on DHPS and promoting eIF5A hypusination. Conversely, in NRAS-mutant melanomas, where pathway activation is more dynamic and subject to feedback regulation, the ERK-DHPS interaction may fluctuate in response to upstream signaling. While direct comparative studies across these genetic backgrounds are currently lacking, emerging evidence suggests that polyamine metabolism and eIF5A hypusination are functionally relevant in both BRAF- and NRAS-driven melanomas [60].

Critically, the dynamic nature of this scaffolding interaction raises important considerations for clinical application of MAPK pathway inhibitors. Current standard-of-care BRAF inhibitors (e.g., vemurafenib, dabrafenib) and MEK inhibitors (e.g., trametinib) suppress ERK phosphorylation [61,62,63], which would be expected to stabilize the ERK-DHPS inhibitory complex and reduce eIF5A hypusination. This effect may contribute to the overall anti-proliferative response to MAPK pathway blockade. However, in the context of acquired resistance—often mediated by reactivation of ERK signaling—the dissociation of ERK from DHPS may be restored, allowing hypusination to resume and potentially facilitating survival of resistant clones. Understanding whether combining MAPK inhibitors with DHPS allosteric inhibitors can prevent or overcome such resistance represents a compelling avenue for future investigation.

#### 2.3.3. The Dual Role of p53 in Stress and Surveillance

The tumor suppressor p53 engages with the hypusination circuit in a context-dependent manner. Under basal conditions, p53 can repress polyamine synthesis, indirectly limiting substrate availability for hypusination [53]. However, under cellular stress (e.g., DNA damage), a p53-dependent program is activated to maintain eIF5A hypusination levels [64,65]. This functional eIF5A is required for the elevated protein synthesis characteristic of cellular senescence, particularly the synthesis of mitochondrial ribosomal proteins, which is crucial for metabolic adaptation. Furthermore, hypusinated eIF5A is necessary for the efficient translation of p53 itself in response to genotoxic stress. Thus, p53 and eIF5A hypusination are linked in a regulatory network that influences cell fate decisions-apoptosis and senescence—and impacts immune surveillance of pre-malignant cells [64,66].

**Figure 2:**
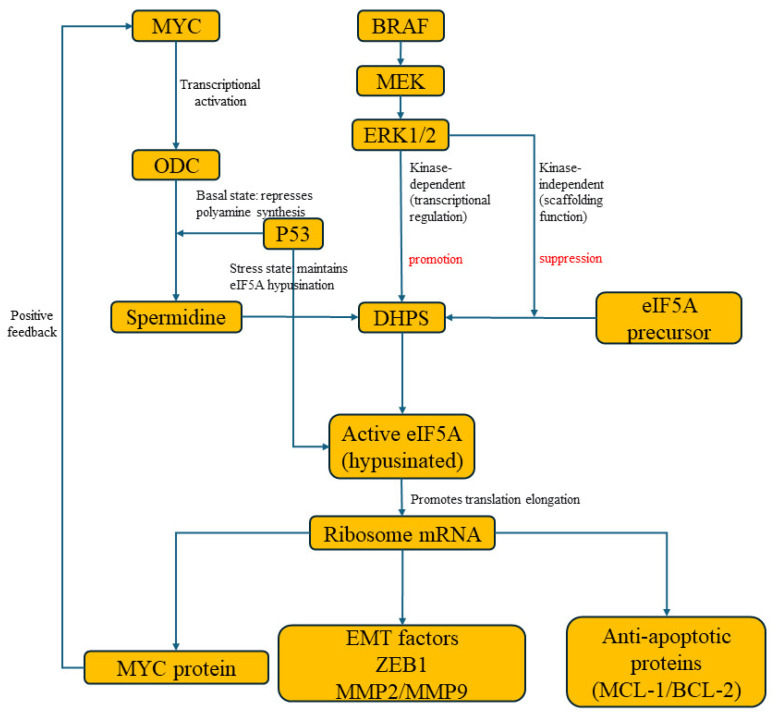
Schematic overview of upstream signaling convergence on the DHPS/eIF5A hypusination axis. MYC drives ODC expression, increasing spermidine availability to fuel eIF5A hypusination. Hypusinated eIF5A reciprocally promotes MYC translation, establishing a positive feed-forward loop. ERK1/2 regulates the axis through both kinase-dependent transcriptional effects and kinase-independent scaffolding interactions. p53 exerts context-dependent effects, repressing polyamine synthesis under basal conditions while maintaining eIF5A hypusination under stress.

In summary, the DHPS/eIF5A axis serves as a convergent signaling hub, translating inputs from major oncogenic pathways [67,68] (MYC, MAPK) and tumor suppressors [69] (p53) into specific translational programs. This integration enables cancer cells to couple their growth signal reception with the precise protein synthesis machinery needed for proliferation, stress adaptation, survival, and evasion of immune destruction (Figure 2).

## 3. The Multifaceted Role of the DHPS/eIF5A Axis in Melanoma Pathogenesis

A key observation supporting the potential selectivity of DHPS inhibition in melanoma comes from comparative analyses across different cell lines. In our previous work, we evaluated the anti-proliferative activity of a series of allosteric DHPS inhibitors against human melanoma cell lines (A375, SK-MEL-28), a murine melanoma cell line (B16), and normal human keratinocytes (HaCaT). Notably, these compounds exhibited potent inhibitory effects on human melanoma cells, while showing considerably weaker activity against B16 cells. Moreover, approximately half of the tested compounds displayed no measurable cytotoxicity against HaCaT cells, strongly indicating a favorable selectivity profile toward malignant cells. Consistent with these phenotypic observations, further analysis revealed marked differences in DHPS expression levels across cell lines. Specifically, A375 and SK-MEL-28 cells expressed high levels of DHPS, whereas B16 cells exhibited substantially lower DHPS abundance compared to human melanoma cells. Importantly, other cancer cell lines—including A549 (lung adenocarcinoma), SW620 (colorectal carcinoma), and MDA-MB-231 (breast carcinoma)—expressed DHPS at levels far below those observed in human melanoma cells. Collectively, these findings provide a mechanistic rationale for the enhanced susceptibility of human melanoma cells to DHPS allosteric inhibition and suggest that the therapeutic window of these inhibitors may be particularly favorable in this malignancy [28].

### 3.1. Fueling the Metastatic Cascade: More than Just Proliferation

The lethality of melanoma is predominantly driven by its metastatic spread, a complex cascade in which the DHPS/eIF5A hypusination circuit plays a fundamental and multi-faceted role [70]. By enabling the efficient translation of pro-migratory proteins, including regulators of the Rho GTPase pathway and actin-binding proteins, this axis directly fuels the motility and invasive capacity of melanoma cells, with experimental inhibition effectively blocking migration and invasion in vitro [30].

Beyond motility, the pathway is critical for sustaining the epithelial-to-mesenchymal transition (EMT) and the associated cellular plasticity required for dissemination. It supports the synthesis of key EMT-transcription factors (e.g., ZEB1) and extracellular matrix-remodeling enzymes such as matrix metalloproteinases (MMP2, MMP9), thereby maintaining the mesenchymal, invasive phenotype [28].

Furthermore, in highly aggressive melanomas capable of forming vasculogenic mimicry (VM)—networks of endothelial-like, fluid-conducting channels that bypass angiogenesis—the hypusination circuit is indispensable. Genetic or pharmacological disruption of DHPS/eIF5A signaling dismantles these VM networks, at least in part by downregulating essential mediators like FGFR2 and c-KIT, highlighting its direct contribution to this alternative perfusion strategy.

### 3.2. Mastering the Art of Stress Resistance

The hypusination circuit is a critical determinant of melanoma cell resilience within the stressful tumor microenvironment. It equips tumor cells to manage proteotoxic stress, as hypusinated eIF5A facilitates the translation of key mediators involved in the unfolded protein response (UPR), and its inhibition can compromise this adaptive program [66]. Furthermore, this pathway intersects with core energy metabolism, influencing mitochondrial function and potentially altering cellular sensitivity to metabolic perturbations [71]. Most critically, the DHPS/eIF5A axis is a key contributor to therapy resistance. By supporting the efficient synthesis of anti-apoptotic proteins (e.g., MCL-1, BCL-2) and DNA repair factors, it enhances cell survival following cytotoxic insult [28]. This mechanism underpins resistance to diverse agents, including targeted BRAF/MEK inhibitors and conventional chemotherapy, highlighting its role as a broad-spectrum survival pathway.

### 3.3. Sculpting an Immunosuppressive Niche: Open Questions and a Dual-Cell Hypothesis

The role of the DHPS/eIF5A axis in shaping the immunosuppressive tumor microenvironment (TME) remains an emerging and incompletely defined area, particularly in melanoma. While the broader polyamine metabolism is well-established as a mediator of immune evasion—impairing T-cell function and promoting the polarization of myeloid-derived suppressor cells (MDSCs) and M2-like tumor-associated macrophages [72,73]—the specific contribution of the hypusination circuit to these processes is less understood. Critically, it remains unresolved whether therapeutic targeting of DHPS/eIF5A primarily exerts its immunomodulatory effects through a cell-autonomous action on melanoma cells, by directly affecting infiltrating immune cells, or through a combination of both.

From a tumor cell-intrinsic perspective, DHPS inhibition may enhance anti-tumor immunity indirectly by inducing immunogenic cell stress or apoptosis in melanoma cells. Preclinical studies in melanoma models have shown that allosteric DHPS inhibitors trigger caspase activation and reduce the expression of pro-survival proteins, events that can increase tumor antigen presentation and sensitivity to T-cell-mediated killing [28,29]. This mechanism does not require the inhibitor to directly act on immune cells; rather, it converts the tumor itself into a more immunogenic target.

Conversely, emerging evidence from non-melanoma models suggests that hypusination is also functionally relevant in immune cells. For instance, eIF5A activity has been implicated in the proliferation and effector function of T cells [66], and polyamine depletion strategies have been shown to reverse T-cell exhaustion and reduce MDSC accumulation in the TME [72]. However, direct evidence demonstrating that DHPS/eIF5A inhibition within immune cells contributes to anti-tumor immunity in melanoma is currently lacking. Whether the available DHPS inhibitors can effectively penetrate and modulate the hypusination status of tumor-infiltrating lymphocytes or myeloid cells remains an open question.

Thus, the proposed dual-mechanism strategy—direct tumor cytotoxicity coupled with immune potentiation—remains a compelling but largely untested hypothesis in melanoma. Future studies using conditional knockout mouse models (e.g., cell-type-specific deletion of *Dhps* in melanoma cells versus immune compartments), along with syngeneic melanoma models treated with allosteric DHPS inhibitors, will be essential to dissect the relative contributions of tumor cell-intrinsic versus immune cell-intrinsic hypusination to the overall anti-tumor response.

## 4. Therapeutic Targeting: From Spermidine Mimetics to Allosteric Warfare

### 4.1. The First Generation: GC7 and Its Limitations

The classic approach to DHPS inhibition has relied on spermidine mimetics. These compounds act by competitively occupying the spermidine-binding site of DHPS, thereby physically blocking the transfer of the 4-aminobutyl moiety to its substrate, the eIF5A precursor protein.

Structurally, these inhibitors typically feature two polar groups, often guanidino or amino functionalities, connected by an extended aliphatic linker, mirroring the architecture of spermidine itself [74,75,76]. Among these, the most extensively studied is GC7 (N^1^-guanyl-1,7-diaminoheptane) [77]. GC7 has served as a vital proof-of-concept molecule, demonstrating potent anti-proliferative activity across a broad spectrum of cancer cell lines in vitro, including HeLa cervical carcinoma [78], N2a neuroblastoma [79], DS19 murine erythroleukemia [79], Tm5 murine melanoma [80], and various human neuroblastoma and head-and-neck squamous carcinoma cells [34]. It has also shown anti-melanoma activity in preclinical in vivo models. However, its clinical translation has been hampered by significant limitations. Primarily, its close structural resemblance to spermidine compromises selectivity, allowing it to potentially interfere with other polyamine-dependent physiological processes. Furthermore, suboptimal pharmacokinetic properties and the ability of cancer cells to compensate by upregulating polyamine transport systems have diminished its efficacy as a standalone therapeutic agent [73,81,82].

While GC7 has demonstrated broad antiproliferative activity across multiple cancer cell lines, its therapeutic window is inherently constrained by the essential nature of DHPS for cell viability [77,83]. Because hypusinated eIF5A is required for proliferation in all eukaryotic cells, inhibition of DHPS would be expected to impair growth in both malignant and non-transformed cells. Indeed, studies have shown that GC7 inhibits the proliferation of non-transformed cells such as NIH3T3 fibroblasts and CHO-K1 cells at concentrations comparable to those effective against cancer cells [78], suggesting a narrow therapeutic index. Moreover, the compound’s structural similarity to spermidine raises concerns regarding off-target effects on other polyamine-dependent processes, including autophagy, ion channel regulation, and oxidative stress responses [84]. These observations underscore the need for more selective inhibitors, such as the allosteric compounds described in Section 4.2, which offer the potential for improved selectivity by targeting a unique DHPS-specific regulatory site rather than competing with spermidine.

### 4.2. The Allosteric Revolution

The paradigm of DHPS inhibition was revolutionized by the structural elucidation of a novel allosteric site, distinct from the catalytic center. This breakthrough stemmed from the work of Tanaka et al., who, through high-throughput screening and subsequent optimization, identified the first-in-class allosteric DHPS inhibitors [85]. Their initial discovery centered on a bromobenzothiophene derivative, compound **11g**, which exhibited potent inhibitory activity. Critically, X-ray crystallographic analysis of the DHPS-**11g** complex revealed a dramatic conformational change in the enzyme, providing definitive evidence for a previously unknown allosteric regulatory site and a non-competitive mechanism of action relative to spermidine.

**Figure 3:**
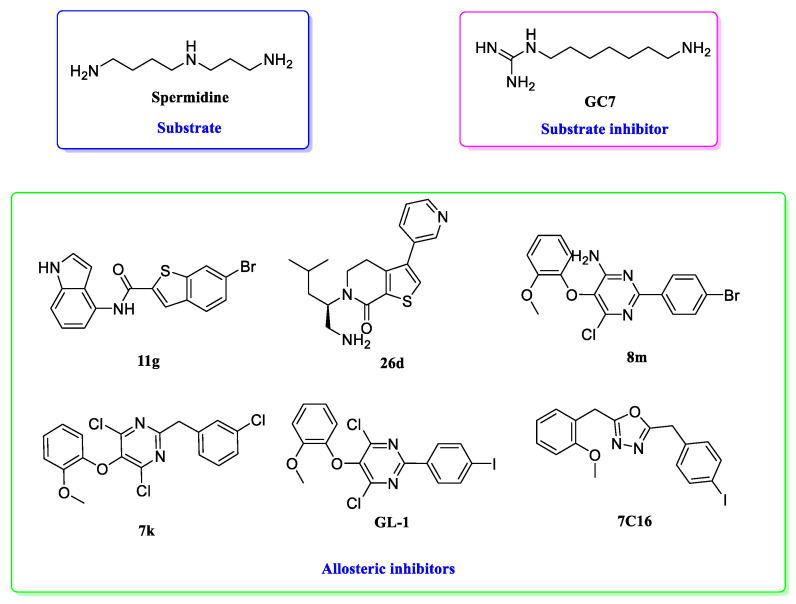
Chemical structures of the substrate, substrate inhibitor, and allosteric inhibitor of DHPS.

Building on this foundational discovery, the same research group subsequently developed a new chemical series based on a fused-ring scaffold, leading to the potent 5,6-dihydrothieno [2,3-c]pyridine derivative (compound **26d**) [86]. Structural studies confirmed that this later inhibitor also binds to the allosteric site but exhibits a distinct binding mode compared to compound **11g**. These seminal studies demonstrated that targeting the allosteric pocket could lock DHPS in an inactive conformation through induced structural changes, establishing a pioneering therapeutic strategy beyond spermidine mimetics (Figure 3).

From a drug safety perspective, the distinction between substrate-mimetic and allosteric inhibitors carries fundamental implications for selectivity. Classical inhibitors such as GC7 are designed to mimic spermidine, featuring two polar groups (guanidino or amino functionalities) connected by an aliphatic linker. While this structural mimicry enables competitive occupation of the spermidine-binding pocket, it also inevitably allows these compounds to interact with other spermidine-dependent processes. Spermidine, beyond serving as the substrate for eIF5A hypusination, participates in numerous essential cellular functions including autophagy regulation [87,88], oxidative stress responses [89,90], ion channel modulation [91], and nucleic acid stabilization [92]. Consequently, GC7 and related spermidine mimetics may interfere with these diverse physiological pathways, contributing to off-target effects and limiting their therapeutic window.

**Figure 4:**
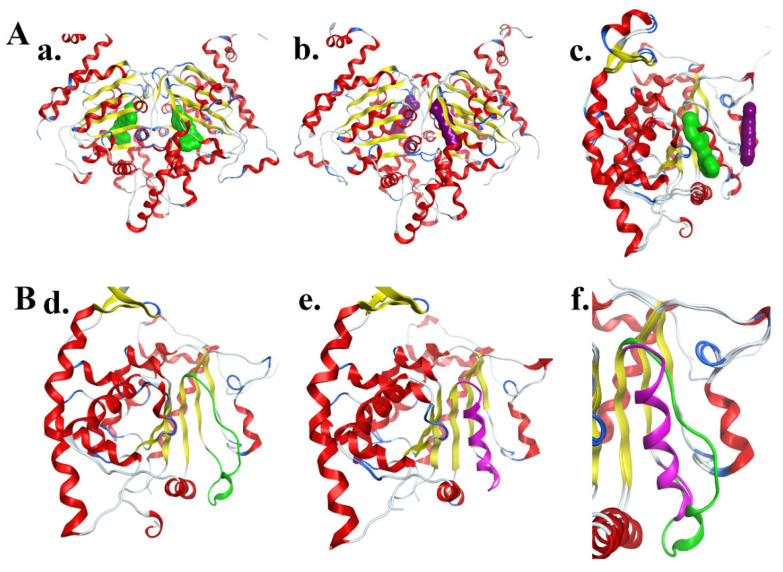
(**A**) Binding sites of the allosteric inhibitor and spermidine in DHPS. (**a**) Binding site of the allosteric inhibitor (shown in green, PDB ID: 6PGR). (**b**) Binding site of the substrate inhibitor (shown in purple, PDB ID: 6P4V). (**c**) Superposition of different DHPS conformations, showing that the allosteric inhibitor binding site is located in close proximity to the substrate inhibitor binding site. (**B**) Conformational changes in DHPS upon binding of different types of inhibitors. (**d**) Binding of the allosteric inhibitor induces unwinding of the α-helix near the binding site. (**e**) Binding of the substrate inhibitor does not induce unwinding of the α-helix. (**f**) Following unwinding, the spatial arrangement of the amino acid residues is altered, allowing them to extend into the spermidine-binding pocket, thereby affecting the catalytic activity of DHPS.

In contrast, allosteric inhibitors such as 11g, 26d bind to a unique pocket located at the DHPS dimer interface—a structural feature distinct from the catalytic site and not shared by other spermidine-binding proteins. Their mechanism of action relies on inducing a conformational change (α-helix unwinding) that is specific to DHPS [85], rather than competing with spermidine for a common binding motif. This allosteric mode of inhibition thus offers the theoretical advantage of greater selectivity, as it minimizes unintended interference with other spermidine-dependent biological processes. While rigorous selectivity profiling across the polyamine interactome remains to be fully established, the distinct binding mode of these allosteric inhibitors provides a rational basis for expecting a more favorable safety profile compared with first-generation substrate mimetics (Figure 4).

This pivotal work laid the essential structural and mechanistic groundwork for the subsequent development of other potent allosteric inhibitors in our work, such as the pyrimidine derivative **8m** [28], **7k**, **GL-1** [29], and the oxadiazole derivative **7C16** [30]. By virtue of their unique mechanism, these allosteric inhibitors, which do not compete with spermidine, offer a promising avenue for combination therapies with polyamine depletion agents (e.g., DFMO) or transport inhibitors, potentially enhancing efficacy and therapeutic index. 

### 4.3. Rational Combination Strategies for Melanoma

The therapeutic potential of targeting the DHPS/eIF5A axis is greatly enhanced when integrated into rational combination regimens, leveraging its unique mechanism to overcome common resistance pathways in melanoma. One highly relevant strategy is co-administration with MAPK pathway inhibitors. While BRAF and MEK inhibitors (BRAFi/MEKi) are standard-of-care for BRAF-mutant melanoma, their efficacy is often limited by cytostatic responses and acquired resistance [93,94]. The addition of a DHPS inhibitor presents a mechanistically coherent approach to convert cytostasis into apoptosis. This is underpinned by the non-canonical, kinase-independent regulation of DHPS-eIF5A interaction by ERK1/2, which links MAPK signaling directly to hypusination efficiency [60]. By inhibiting the synthesis of short-lived pro-survival proteins (e.g., MCL-1) whose translation is maintained by oncogenic signaling and facilitated by eIF5A, DHPS blockade can deplete this critical survival buffer and potentiate the cytotoxic effects of MAPK inhibition [28,66].

A second paradigm involves combination with immunotherapies, aiming to remodel the immunosuppressive TME. Elevated polyamine levels contribute to an immune-cold TME by inhibiting T-cell function and promoting the activity of MDSCs [72,73]. Preclinical evidence demonstrates that depleting polyamines via a combination of the biosynthesis inhibitor DFMO and a polyamine transport inhibitor can reverse this immunosuppression and synergize with immune checkpoint blockade [73]. Incorporating a DHPS inhibitor could provide a dual attack: first, by directly inducing immunogenic stress and apoptosis in tumor cells, thereby potentially enhancing antigen presentation and T-cell priming [71,95]. And second, by depleting the spermidine substrate required for hypusination, it may further disrupt the functional adaptation of both tumor and immunosuppressive cells within the TME, creating a more favorable context for immune-mediated tumor clearance [72,73].

Finally, combining DHPS/eIF5A inhibition with genotoxic chemotherapeutic agents or radiotherapy represents a logical approach to overcome treatment resistance. The efficacy of DNA-damaging therapies is frequently limited by the tumor’s capacity to rapidly deploy DNA repair machinery. The hypusination circuit is essential for the efficient translation of a subset of proteins, including those involved in the DNA damage response and repair pathways [48,96]. By impairing the de novo synthesis of key DNA repair enzymes, DHPS inhibition could prevent the resolution of therapy-induced DNA lesions, thereby sensitizing melanoma cells to a broad range of genotoxic insults [82,97]. This strategy targets a fundamental vulnerability—the tumor’s reliance on rapid protein synthesis for adaptive survival—and could help mitigate a common cause of therapeutic failure.

While these combination strategies offer compelling rationale, their clinical translation must consider several critical factors. First, additive toxicities may arise from overlapping requirements for eIF5A-mediated translation in rapidly dividing normal tissues, particularly hematopoietic and gastrointestinal cells [83,84]. Careful dose optimization and intermittent scheduling will therefore be essential. Second, the tumor-selective synergy of these combinations likely stems from the heightened dependency of melanoma cells on DHPS/eIF5A—driven by constitutive MAPK signaling and MYC activation—coupled with their elevated DHPS expression compared to normal cells [28,58,60]. This differential reliance creates a therapeutic window. Third, the impact on immune function is complex: while activated T cells require eIF5A for proliferation and effector function, polyamine depletion has been shown to preferentially target immunosuppressive populations such as MDSCs and M2 macrophages [72,73]. The net effect of DHPS inhibition on anti-tumor immunity in melanoma thus remains an open question requiring rigorous evaluation in immunocompetent models.

## 5. Challenges, Future Perspectives, and Translational Roadmap

The translation of DHPS/eIF5A inhibition into clinical practice faces several key challenges that define critical future research directions. A foremost hurdle is patient stratification, as not all melanomas exhibit equal dependence on this pathway. The identification and validation of predictive biomarkers are therefore essential for enriching clinical trials with likely responders. Candidate biomarkers include elevated expression of DHPS or its specific substrate isoform eIF5A2, increased levels of hypusinated eIF5A in tumor tissue, or specific genomic backgrounds such as concurrent BRAF mutation and PTEN loss that may confer heightened addiction to the hypusination circuit [98]. Implementing such biomarker-driven strategies will be crucial for optimizing therapeutic efficacy and trial design (Table 1).

Another significant challenge lies in managing the therapeutic index, given the fundamental role of hypusination in normal cellular physiology, particularly in neurodevelopment. Systemic, continuous inhibition carries a risk of on-target toxicity [83,84]. To mitigate this, innovative drug delivery and dosing strategies are needed. These may include the development of nanoparticle-based carriers for tumor-targeted delivery, intermittent dosing regimens designed to exploit the differential addiction between rapidly proliferating tumor cells and most normal tissues, or topical formulations for the treatment of primary or cutaneous metastatic melanoma to minimize systemic exposure [71,99] (Table 1).

Looking beyond direct enzyme inhibition, a deeper understanding of the pathway’s downstream effectors could unveil new therapeutic opportunities. Future research should aim to comprehensively define the specific suite of pro-tumorigenic proteins in melanoma whose translation is critically dependent on hypusinated eIF5A. This “hypusinome” profiling could reveal more druggable, and potentially more tissue-specific, downstream targets than DHPS itself, offering alternative intervention points with potentially improved safety profiles [100,101].

Finally, to fully grasp the therapeutic vulnerability, the DHPS/eIF5A axis must be studied within the integrated context of melanoma biology. Future investigations need to elucidate how extracellular nutrient inputs—such as glutamine and arginine that feed into polyamine synthesis—and oncogenic signals converge to regulate this pathway [12]. Furthermore, understanding its interaction with other major translational control nodes, including the mTORC1 and eIF4F complexes, will reveal potential compensatory mechanisms and rational combination strategies, painting a complete picture of its role in the malignant translation program [39,96].

## 6. Conclusions

The DHPS/eIF5A hypusination pathway represents a formidable vulnerability in melanoma, acting as a central processor that converts metabolic resources (polyamines) and oncogenic signals into a specialized translational output that fuels every facet of the disease—from uncontrolled growth and invasion to microenvironment manipulation. Moving beyond the era of broad polyamine depletion, the development of precise allosteric DHPS inhibitors heralds a new therapeutic modality. By crippling the tumor’s ability to synthesize its malignant proteome, these agents hold the potential to undermine melanoma’s adaptability and resilience. As we stand at the confluence of cancer metabolism, translation, and immunotherapy, targeting this ancient and unique modification pathway offers a promising and novel strategy to combat one of the most aggressive human cancers.

## Figures and Tables

**Table 1:** Summary of DHPS inhibitors and their development status in melanoma.

Inhibitor	Class	Development Stage	Notes
GC7	Substrate mimetic	Preclinical/Exploratory	Broad antiproliferative activity; limited by poor selectivity and off-target effects due to interference with other spermidine-dependent processes
11g	Allosteric inhibitor	Preclinical	First-in-class allosteric inhibitor; induces conformational change in DHPS; validated in biochemical and structural studies
26d	Allosteric inhibitor	Preclinical	Fused-ring scaffold with distinct binding mode compared to 11g; potent inhibitory activity
8m	Allosteric inhibitor	Preclinical	Pyrimidine derivative; potent anti-melanoma activity in vitro and in vivo; activates caspase-3
7k	Allosteric inhibitor	Preclinical	Suppresses vasculogenic mimicry (VM) via downregulation of FGFR2 and c-KIT; favorable pharmacokinetic profile
GL-1	Allosteric inhibitor	Preclinical	Inhibits DHPS-eIF5A binding; promotes Cu^2+^ accumulation-induced apoptosis; regulates METTL3 m^6^A modification
7C16	Allosteric inhibitor	Preclinical	Oxadiazole derivative; inhibits melanoma cell migration and invasion; active in zebrafish xenograft models

## Data Availability

No new data were created or analyzed in this study.
